# Supplementary material for: Fabrication of Active Z-Scheme Sr2MgSi2O7: Eu2+, Dy3+/COF Photocatalyst for Round-the-Clock Efficient Removal of Total Cr
Source: Molecules. 2024 Sep 12;29(18):4327. doi: 10.3390/molecules29184327 (PMC11434180; doi:10.3390/molecules29184327)
Supplement: Supplementary file 1 [file molecules-29-04327-s001.zip › molecules-3142880-supplementary.pdf]

Electronic Supporting Information (ESI)

Fabrication of Active Z-Scheme $Sr_2MgSi_2O_7$: Eu^{2+}, Dy^{3+}/COF Photocatalyst for Round-the-Clock Efficient Removal of Total Cr

Meng Xu, Junshu Wu, Mupeng Zheng * and Jinshu Wang *

Key Laboratory of Advanced Functional Materials, Ministry of Education, College of Materials Science & Engineering, Beijing University of Technology, Beijing 100124, China; xum11062020@163.com (M.X.); junshuwu@bjut.edu.cn (J.W.)

* Correspondence: mpzheng@bjut.edu.cn (M.Z.); wangjsh@bjut.edu.cn (J.W.); Tel.: +86-10-6739-1101 (J.W.)

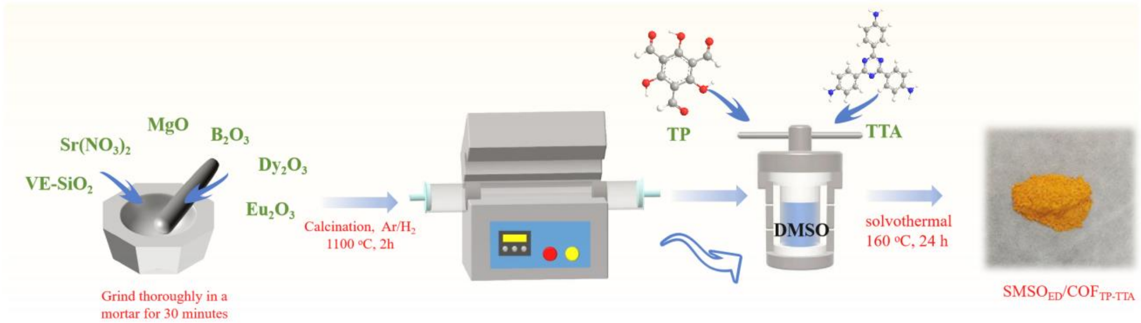

Figure S1. Schematic illustration for the preparation of $SMSO_{ED}$ and $SMSO_{ED}/COF_{TP\text{-}TTA}$.

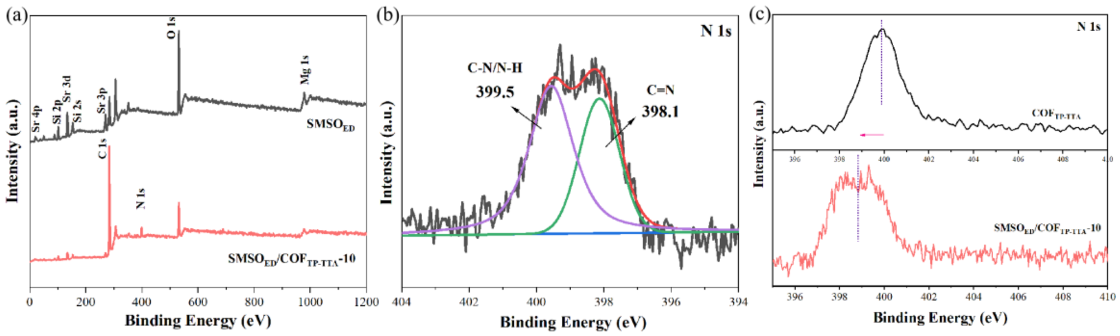

Figure S2. (a) The XPS survey scan of $SMSO_{ED}$ and $SMSO_{ED}/COF_{TP-TTA}$-10. (b) N1s regions of $SMSO_{ED}/COF_{TP-TTA}$-10. (c) N1s regions of COF_{TP-TTA}-10 and $SMSO_{ED}/COF_{TP-TTA}$-10.

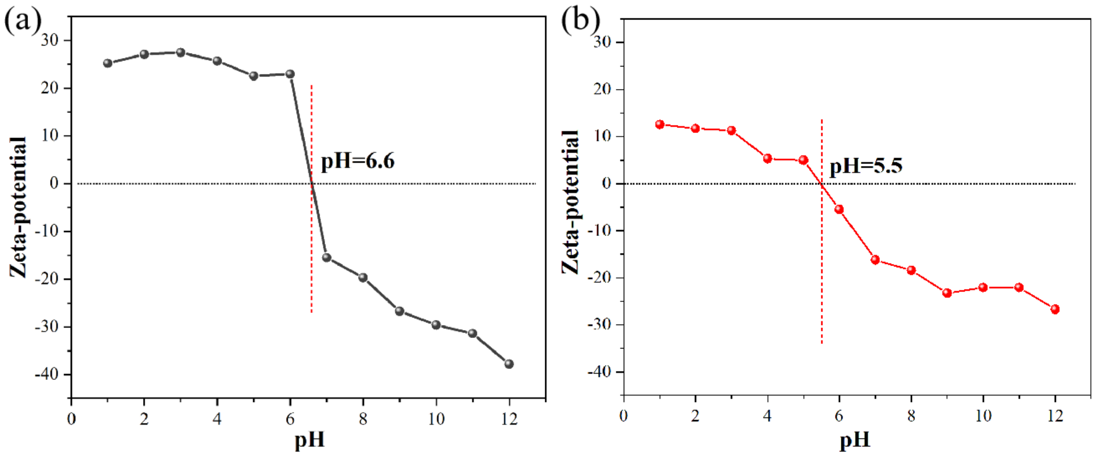

Figure S3. Zeta potentials of (a) $COF_{TP\text{-}TTA}$ and (b) $SMSO_{ED}/COF_{TP\text{-}TTA}$-10 as a function of solution pH value.

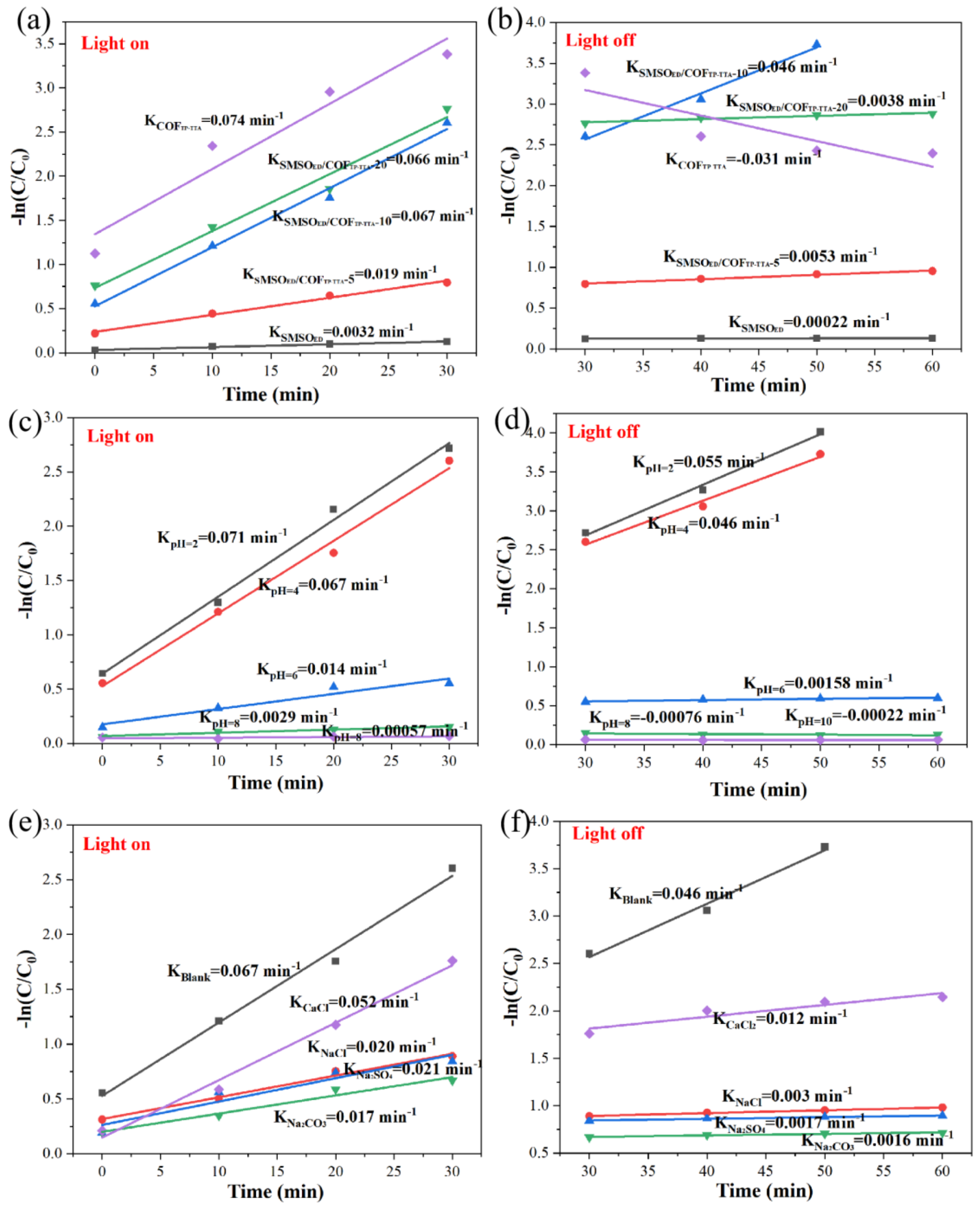

Figure S4. The Cr(VI) reduction kinetic rate constants of $SMSO_{ED}/COF_{TP-TTA}$-10 under different conditions. (Reaction conditions in (a, b): [Cr(VI)] = 10 mg/L, [Catalysts] = 0.5 g/L, pH = 4 and T = 25 ^{o}C; (c, d) pH = 2-10 (e, f) [co-existing ions] = 5 mM)

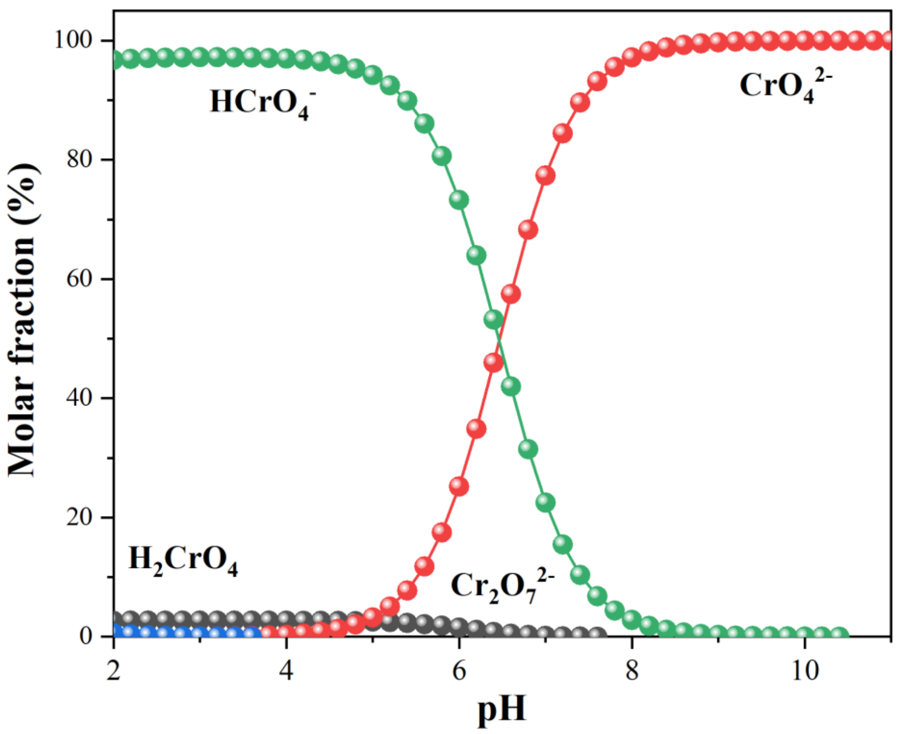

Figure S5. Species distribution of Cr(VI) depending on solution pH value.

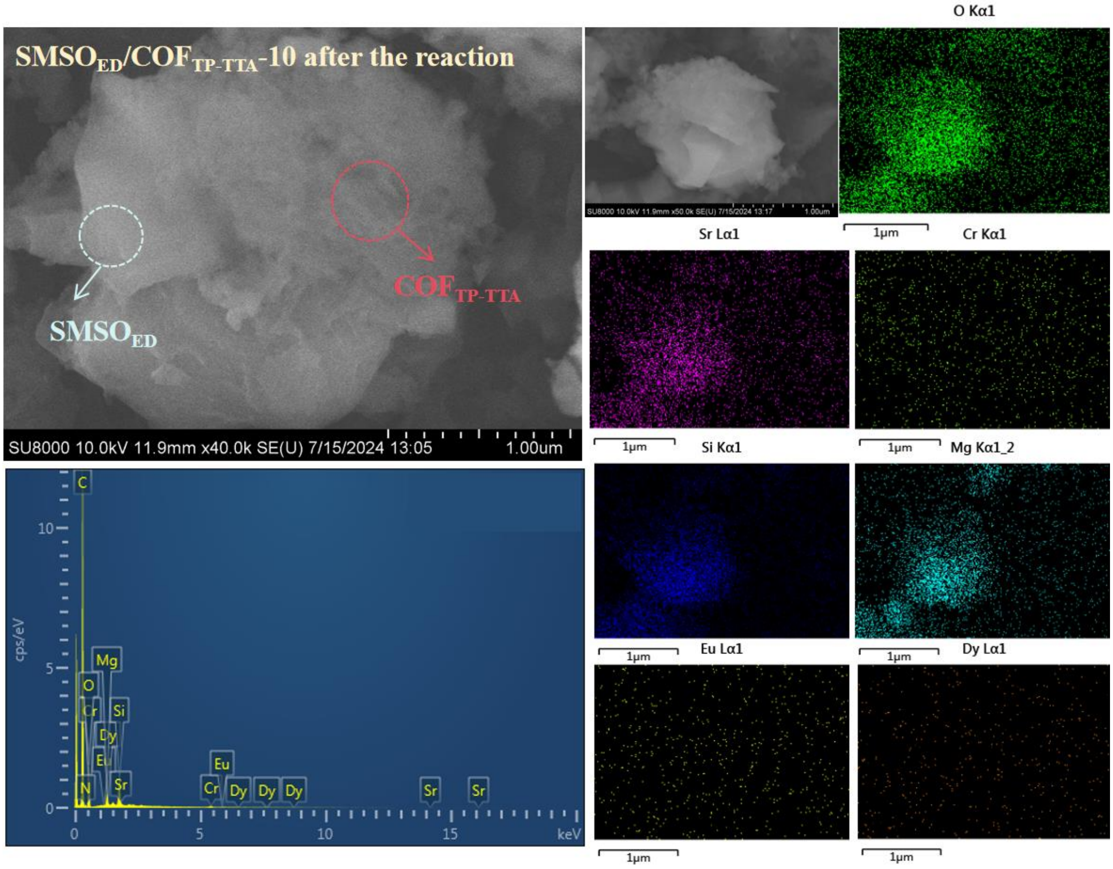

Figure S6. SEM images of SMSO$_{ED}$/COF$_{TP-TTA}$-10 after the Cr(T) removal and the corresponding mapping images.

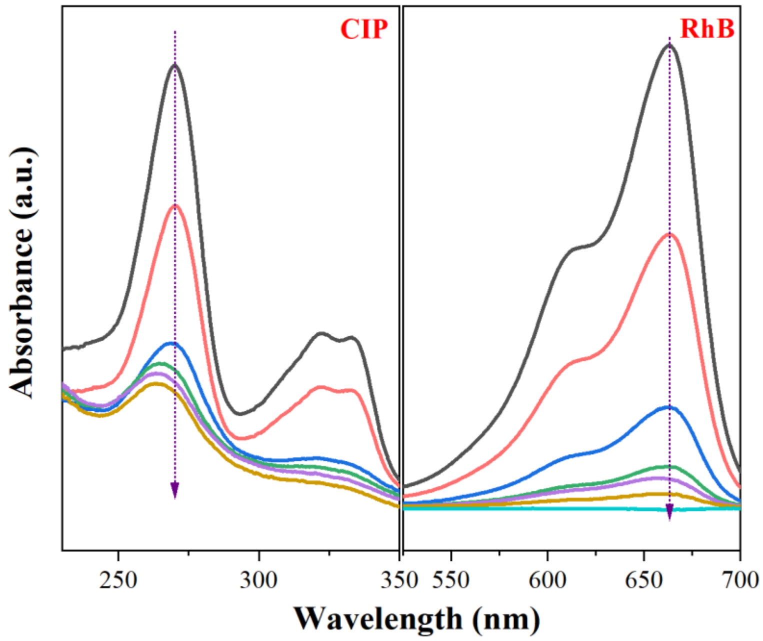

Figure S7. UV-vis absorption spectra of CIP and RhB during photocatalytic degradation over the used $SMSO_{ED}/COF_{TP-TTA}$-10 under light irradiation conditions, showing the reuse of the generated Cr product-loaded $SMSO_{ED}/COF_{TP-TTA}$-10. (Conditions: 30 mgCr product-loaded $SMSO_{ED}/COF_{TP-TTA}$-10, 50 mL 10 mg/L dyes solution, pH without adjust, room temperature).

In general, catalysts containing Cr species are discarded into the environment, causing secondary pollution. Given its powerful ability to generate oxidative free radicals ($O_2^{\cdot-}$ and ·OH), the $SMSO_{ED}/COF_{TP-TTA}$-10 after the reaction can be applied in the degradation of organic pollutants. The results are presented in **Fig. S7**. The data demonstrate that catalysts containing Cr display high photocatalytic activity, which suggests that the $SMSO_{ED}/COF_{TP-TTA}$-10 catalyst is a promising photocatalyst with good reusability.

Table S1. The comparison of removal efficiencies of Cr(T) in the photocatalysis systems

Material	Operation conditions	Cr(VI) reduction efficiencies	Cr(T) removal efficiencies	External conditions	Reference
Leonardite	[material]=0.25 $g \cdot L^{-1}$, $[Cr(VI)]_0$=10 $mg \cdot L^{-1}$, pH=2	100%, 2 h	100%	Under irradiation	[1]
MXene	[material]=1.5 $g \cdot L^{-1}$, $[Cr(VI)]_0$=5$mg \cdot L^{-1}$, pH_0=4	100%, 3 h	100%	Under irradiation	[2]
ZIF-8@TiO_2	[material]=1 $g \cdot L^{-1}$, $[Cr(VI)]_0$=20 $mg \cdot L^{-1}$, pH_0=7	93.1%, 2 h	-	Under irradiation	[3]
acid-modified g-C_3N_4	[material]=1 $g \cdot L^{-1}$, $[Cr(VI)]_0$=40 $mg \cdot L^{-1}$, pH_0=2	99.7%, 1.25 h	-	Under irradiation	[4]
$SMSO_{ED}/COF_{TP-TTA}$-10	[material]=0.5 $g \cdot L^{-1}$, $[Cr(VI)]_0$=10 $mg \cdot L^{-1}$, pH_0=4	100%, 1 h	100%	Round-the-clock	This work

Reference

[53] Arslan, H., Eskikaya, O., Bilici, Z., Dizge, N., Balakrishnan, D. (2022). Comparison of Cr (VI) adsorption and photocatalytic reduction efficiency using leonardite powder. Chemosphere, 300, 134492.

[54] Jamaluddin, N. S., Alias, N. H., Samitsu, S., Othman, N. H., Jaafar, J., Marpani, F., Tan, Y. Z. (2022). Efficient chromium (VI) removal from wastewater by adsorption-assisted photocatalysis using MXene. Journal of Environmental Chemical Engineering, 10(6), 108665.

[55] Song, Y., Lu, X., Liu, Z., Liu, W., Gai, L., Gao, X., Ma, H. (2022). Efficient removal of Cr (VI) by TiO2 based micro-nano reactor via the synergy of adsorption and photocatalysis. Nanomaterials, 12(2), 291.

[56] Sun, H., Wang, L., Liu, Y., Cheng, Z., Zhao, Y., Guo, H., Yin, X. (2022). Photocatalytic reduction of Cr (VI) via surface modified g-C3N4 by acid-base regulation. Journal of Environmental Management, 324, 116431.
